# Supplementary material for: Differential DNA Methylation from Autistic Children Enriches Evidence for Genes Associated with ASD and New Candidate Genes
Source: Brain Sci. 2023 Oct 7;13(10):1420. doi: 10.3390/brainsci13101420 (PMC10605446; doi:10.3390/brainsci13101420)
Supplement: Supplementary file 1 [file brainsci-13-01420-s001.zip › suppl mat/Figure S1.pdf]

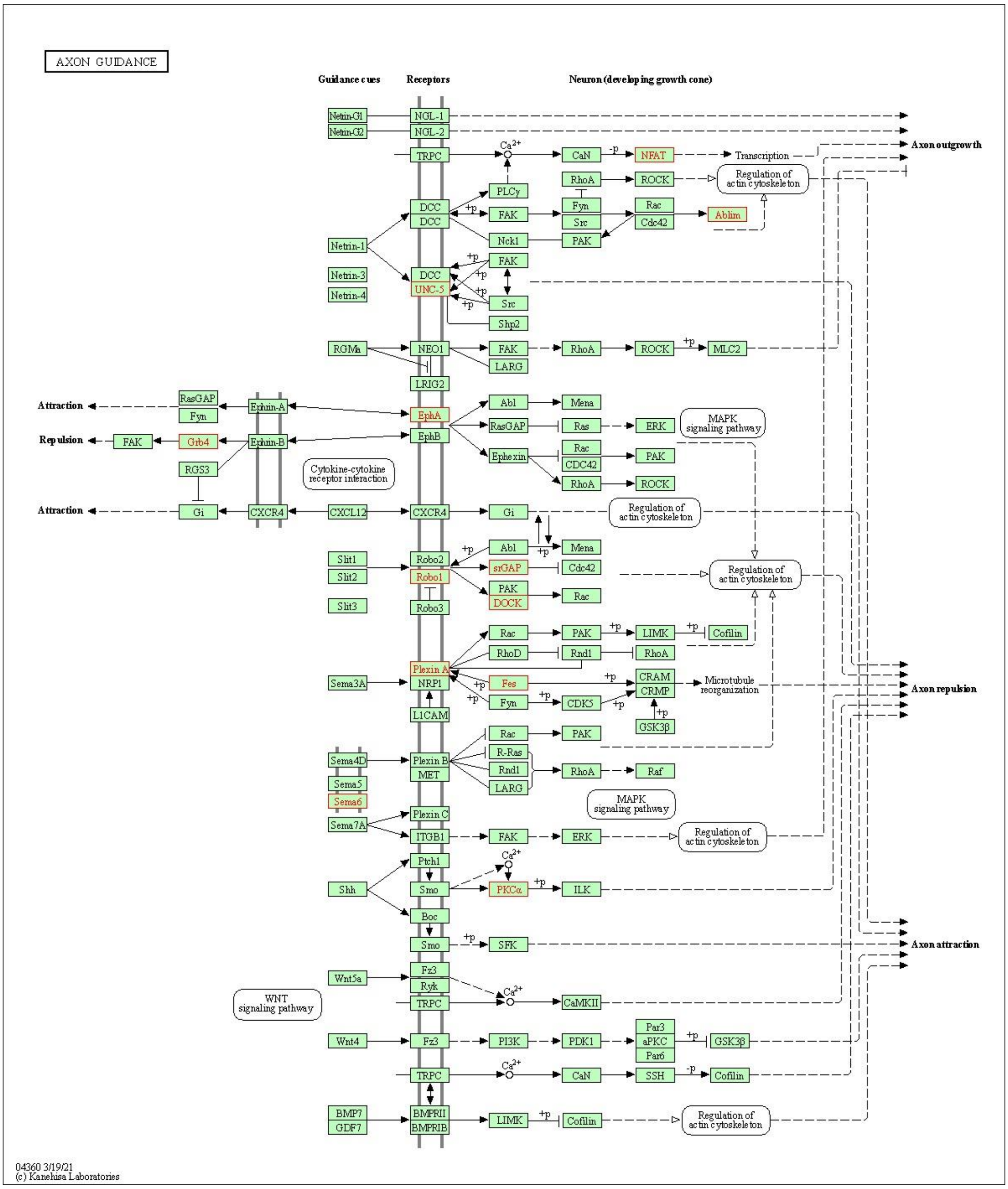

Supplementary Figure S1. Axon guidance pathway from KEGG data base. The genes harboring DMCs are shows in red.
