# Supplementary material for: Differential DNA Methylation from Autistic Children Enriches Evidence for Genes Associated with ASD and New Candidate Genes
Source: Brain Sci. 2023 Oct 7;13(10):1420. doi: 10.3390/brainsci13101420 (PMC10605446; doi:10.3390/brainsci13101420)
Supplement: Supplementary file 1 [file brainsci-13-01420-s001.zip › suppl mat/Table S2.pdf]

Table S2.

Enriched gene set	Gene symbol	Gene name
**Aortic disease**	*AEBP1*	AE binding protein 1
	COL12A1	collagen type XII alpha 1 chain
	COL5A1	collagen type V alpha 1 chain
	CRIP2	cysteine rich protein 2
	CTSZ	cathepsin Z
	FBN1	fibrillin 1
	FOXE3	forkhead box E3
	SPON1	spondin 1
**Premature birth**	*COL5A1*	collagen type V alpha 1 chain
	COL5A2	collagen type V alpha 2 chain
	CYP26C1	cytochrome P450 family 26 subfamily C member 1
	EPHX1	epoxide hydrolase 1
	GSTM1	glutathione S-transferase mu 1
	GSTP1	glutathione S-transferase pi 1
	IGF1	insulin like growth factor 1

Table S2.

	IL10RA	interleukin 10 receptor subunit alpha
	KCNJ1	potassium voltage-gated channel subfamily J member 1
	NDP	NDP, norrin cystine knot growth factor
	PTGER2	prostaglandin E receptor 2
	PTGER3	prostaglandin E receptor 3
	SFTPA1	surfactant protein A1
	TFAP2B	transcription factor AP-2 beta
	TRAPPC2	trafficking protein particle complex 2
	TSHR	thyroid stimulating hormone receptor
**Musculoskeletal diseases**	*AMPD1*	adenosine monophosphate deaminase 1
	ANKRD11	ankyrin repeat domain 11
	ANTXR2	ANTXR cell adhesion molecule 2
	BGLAP	bone gamma-carboxyglutamate protein
	COL5A1	collagen type V alpha 1 chain
	DLX5	distal-less homeobox 5
	DMD	dystrophin

Table S2.

DNAJB6	DnaJ heat shock protein family (Hsp40) member B6
EXT1	exostosin glycosyltransferase 1
EZR	ezrin
FBN1	fibrillin 1
FGFR2	fibroblast growth factor receptor 2
FKRP	fukutin related protein
FRAS1	Fraser extracellular matrix complex subunit 1
HLA-DQA1	major histocompatibility complex, class II, DQ alpha 1
IGF1	insulin like growth factor 1
IL17A	interleukin 17A
KAT6B	lysine acetyltransferase 6B
MBNL2	muscleblind like splicing regulator 2
MTM1	myotubularin 1
NELL1	neural EGFL like 1
PADI4	peptidyl arginine deiminase 4
PAX8	paired box 8
RYR1	ryanodine receptor 1

Table S2.

	SMARCAL1	SWI/SNF related, matrix associated, actin dependent regulator of chromatin, subfamily a like 1
	TBX1	T-box 1
	TNXB	tenascin XB
	TRAPPC2	trafficking protein particle complex 2
	TRIM21	tripartite motif containing 21
	TSHR	thyroid stimulating hormone receptor
